# Intratumoral heterogeneity and *TERT* promoter mutations in progressive/higher-grade meningiomas

**DOI:** 10.18632/oncotarget.22650

**Published:** 2017-11-24

**Authors:** Tareq A. Juratli, Christian Thiede, Mara V.A. Koerner, Shilpa S. Tummala, Dirk Daubner, Ganesh M. Shankar, Erik A. Williams, Maria Martinez-Lage, Silke Soucek, Katja Robel, Tristan Penson, Mechthild Krause, Steffen Appold, Matthias Meinhardt, Thomas Pinzer, Julie J. Miller, Dietmar Krex, Heather A. Ely, Ian M. Silverman, Jason Christiansen, Gabriele Schackert, Hiroaki Wakimoto, Matthias Kirsch, Priscilla K. Brastianos, Daniel P. Cahill

**Affiliations:** ^1^ Translational Neuro-Oncology Laboratory, Department of Neurosurgery, Massachusetts General Hospital Cancer Center, Harvard Medical School, Boston, Massachusetts, USA; ^2^ Department of Neurosurgery, Medizinische Fakultät Carl Gustav Carus, Technische Universität Dresden, Dresden, Germany; ^3^ Department of Medicine I, Medizinische Fakultät Carl Gustav Carus, Technische Universität Dresden, Dresden, Germany; ^4^ Institute of Neuroradiology, Medizinische Fakultät Carl Gustav Carus, Technische Universität Dresden, Dresden, Germany; ^5^ Department of Pathology, Massachusetts General Hospital and Harvard Medical School, Boston, Massachusetts, USA; ^6^ Institute of Radiooncology, Helmholtz-Zentrum Dresden - Rossendorf, Dresden, Germany; ^7^ German Cancer Consortium (DKTK) Dresden and German Cancer Research Center (DKFZ), Heidelberg, Germany; ^8^ Department of Radiation Oncology and OncoRay, Medizinische Fakultät Carl Gustav Carus, Technische Universität Dresden, Dresden, Germany; ^9^ Institute of Pathology, Medizinische Fakultät Carl Gustav Carus, Technische Universität Dresden, Dresden, Germany; ^10^ Department of Neurology, Massachusetts General Hospital and Harvard Medical School, Boston, Massachusetts, USA; ^11^ Ignyta, Inc., San Diego, California, USA; ^12^ Department of Medicine, Department of Neurology, Massachusetts General Hospital and Harvard Medical School, Boston, Massachusetts, USA

**Keywords:** meningioma, telomere, heterogeneity, rearrangements, fusion

## Abstract

**Background:**

Recent studies have reported mutations in the telomerase reverse transcriptase promoter (*TERT*p) in meningiomas. We sought to determine the frequency, clonality and clinical significance of telomere gene alterations in a cohort of patients with progressive/higher-grade meningiomas.

**Methods:**

We characterized 64 temporally- and regionally-distinct specimens from 26 WHO grade III meningioma patients. On initial diagnoses, the meningiomas spanned all WHO grades (3 grade I, 13 grade II and 10 grade III). The tumor samples were screened for *TERT*p and *ATRX/DAXX* mutations, and *TERT* rearrangements. Additionally, *TERT*p was sequenced in a separate cohort of 19 patients with radiation-associated meningiomas. We examined the impact of mutational status on patients’ progression and overall survival.

**Results:**

Somatic *TERT*p mutations were detected in six patients (6/26 = 23%). Regional intratumoral heterogeneity in *TERT*p mutation status was noted. In 4 patients, *TERT*p mutations were detected in recurrent specimens but not in the available specimens of the first surgery. Additionally, a *TERT* gene fusion (*LPCAT1-TERT*) was found in one sample. In contrary, none of the investigated samples harbored an *ATRX* or *DAXX* mutation. In the cohort of radiation-induced meningiomas, *TERT*p mutation was detected in two patients (10.5%). Importantly, we found that patients with emergence of *TERT*p mutations had a substantially shorter OS than their *TERT*p wild-type counterparts (2.7 years, 95% CI 0.9 – 4.5 years versus 10.8 years, 95% CI 7.8 -12.8 years, p=0.003).

**Conclusions:**

In progressive/higher-grade meningiomas,*TERT*p mutations are associated with poor survival, supporting a model in which selection of this alteration is a harbinger of aggressive tumor development. In addition, we observe spatial intratumoral heterogeneity of *TERT*p mutation status, consistent with this model of late emergence in tumor evolution. Thus, early detection of *TERT*p mutations may define patients with more aggressive meningiomas. Stratification for *TERT* alterations should be adopted in future clinical trials of progressive/higher-grade meningiomas.

## INTRODUCTION

Modern genomic technologies have allowed for comprehensive characterization of somatic gene mutations found in tumor cells. In cohorts of newly-diagnosed meningioma, mutations in *AKT1*, *SMO*, *KLF4*, *PIK3CA*, and *TRAF7* have been identified, in addition to the long-established NF2 gene inactivation that is characteristic of this disease [1]. More recently, we and others have identified additional genomic alterations that are specifically associated with progressive higher-grade meningiomas, including BAP1 inactivation [2] and telomerase reverse transcriptase promoter *(TERT*p*)* mutations [3].

Telomere maintenance is considered a hallmark of neoplasia [4]. Over 90% of human tumors express the enzyme telomerase, which actively counteracts telomere shortening [5]. *TERT*p mutations, initially discovered in melanomas [6], are among the most common recurrent alterations in human cancer. In addition to *TERT*p mutations, aberrant telomerase expression can be caused by *TERT* rearrangements [7], DNA amplifications or transcript fusions [5]. Telomerase-independent cancers maintain their telomeres through a homologous recombination-dependent process known as alternative lengthening of telomeres (ALT), which is associated with genomic alterations in the genes *DAXX* or *ATRX* [5].

Telomerase activation has been reported as a frequent phenomenon in meningiomas, with an association between activation and World Health Organization (WHO) grade [3]. Intriguingly, *TERT*p mutations were reported to be rare in WHO grades I and II meningiomas [8–10], the vast majority of which are cured by surgical resection, but were found at higher frequencies (up to 28%) in recurrent tumors and those with malignant histopathological appearance, which is predictive of a higher recurrence rate [3, 8].

However, a longitudinal genomic analysis of telomerase gene alterations across the spectrum of initial and recurrent disease remains incomplete. It is possible that other known cancer-specific mechanisms of telomere maintenance such as *ATRX/DAXX* mutations or *TERT* rearrangements could be present in meningiomas. With this in mind, we characterized a large cohort of patients with progressive/higher-grade meningiomas at both initial diagnosis and recurrence. To investigate the selective pressures that manifest in genomic differences during meningioma progression, we also sequenced temporally and spatially distinct sites of the meningiomas in our cohort to assess for intratumoral heterogeneity.

## RESULTS

### Somatic *TERT*p mutations are frequent in recurrent meningiomas

*TERT*p was sequenced in all 64 tumor samples derived from 26 patients with progressive higher-grade meningiomas, across the temporal spectrum of disease from the initial pre-treatment specimen to the final recurrent tumor. Canonical *TERT*p mutations were detected in nine samples (9/64= 14%) from six patients (three females and three males, 6/26 = 23%). Three *TERT*p mutations were located at 146 bp (referred C250T) and three were located 124 bp (referred C228T) upstream of the translation start (Table 1). We screened DNA from matched blood samples available in four positive cases and did not detect *TERT*p mutations, confirming that this is a somatic (non-germline) alteration, as has previously been reported in other cancers [11].

**Table 1:** Patients’ characteristics

	Gender	Age at first diagnosis	Initial WHO grading	Tumor location	*TERT* promoter status	Nr. of surgeries	RTx	CTx
**Pat01**	f	62	II	Convexity	wild-type	4	Yes	No
**Pat02**	m	73	III	Intraventricular	wild-type	2	Yes	No
**Pat03**	m	48	III	Convexity	wild-type	1	Yes	No
**Pat04**	m	42	III	Convexity	wild-type	1	Yes	No
**Pat05**	f	52	III	Convexity	wild-type	1	No	No
**Pat06**	m	75	III	Convexity	C228T mutation	1	Yes	No
**Pat07**	m	75	II	Frontobasal	C228T mutation	5	Yes	No
**Pat08**	m	65	II	Convexity	C250T mutation	6	Yes	No
**Pat09**	f	64	II	Frontobasal	wild-type	3	Yes	No
**Pat10**	m	50	II	pertoclival	wild-type	3	Yes	Imatinib
**Pat11**	m	54	II	Frontobasal	wild-type	4	Yes	No
**Pat12**	f	49	II	Infratentorial	wild-type	4	Yes	No
**Pat13**	m	65	II	Convexity	wild-type/ *LPCAT1*-*TERT* fusion	8	Yes	No
**Pat14**	m	61	III	Infratentorial	wild-type	3	Yes	No
**Pat15**	m	18	I	Frontobasal	wild-type	4	Yes	Temozolomide
**Pat16**	f	6	II	Temporobasal	wild-type	4	Yes	No
**Pat17**	f	47	III	Convexity	wild-type	1	No	No
**Pat18**	m	50	III	Unknown	wild-type	3	No	No
**Pat19**	m	75	III	Unknown	wild-type	1	No	No
**Pat20**	f	62	III	Frontobasal	wild-type	1	Yes	Hydroxyurea
**Pat21**	f	60	II	Temporobasal	wild-type	4	Yes	No
**Pat22**	m	71	I	Frontobasal	wild-type	2	Yes	No
**Pat23**	m	57	II	Convexity	wild-type	3	Yes	No
**Pat24**	f	74	II	Frontobasal	C250T mutation	2	No	No
**Pat25**	f	45	II	Convexity	C228T mutation	2	Yes	No
**Pat26**	f	47	I	Convexity	C250T mutation	2	Yes^*^	No

M: male, f: female, RTx: radiotherapy, CTx: chemotherapy. WHO: World Health Organization. ^*^ upfront radiotherapy.

### Somatic *TERT*p mutations are acquired during progression in recurrent meningiomas

**Figure 1:**
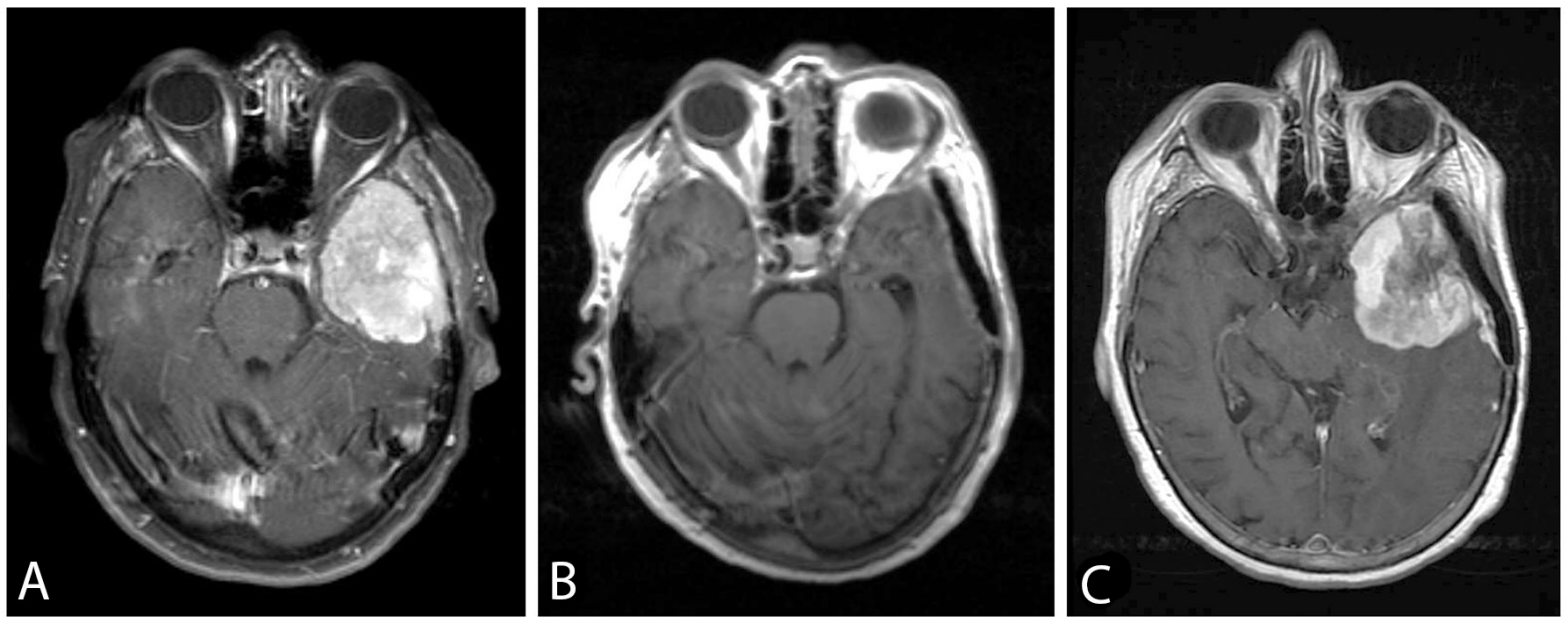
Axial post-gadolinium T1-weighted images showing the *TERTp* wild-type sphenoid wing meningioma WHO grade II (Case Pat24) **(A)** pre- and **(B)** postoperatively. An adjuvant treatment was not pursued after initial resection. **(C)** 22 months later the patient showed a local recurrent tumor. At that time point, the tumor was histologically progressive to grade III and carried a newly detected *TERTp* mutation. The patient had a rapidly progressive subsequent course, and died 6 months after the second surgery.

A matched comparison of primary meningiomas and their recurrences is often challenging due to the long time lapse between initial tumor and recurrence. Our cohort had 19 matched pairs, allowing the determination of timing of acquired mutations during progression. To investigate whether the *TERT*p mutation was acquired at recurrence in our cohort, we examined *TERTp* status longitudinally in samples. Interestingly, out of five *TERT*p-mutant cases with the initial specimen available, four somatic *TERT*p mutations were absent in the initial, grade II tumor, emerging only later in the recurrent tumors (Table 2, Figure 1). On inspection of clinical demographic factors (Table 1), we could not identify differences in the initial presentation between patients who ultimately developed *TERT*p mutations compared to those with *TERT*p wild-type meningiomas. The median age of the patient at first diagnosis was comparable in both groups and we could not discern a correlation between the tumor location and *TERT*p mutation status. These findings highlight the temporal dynamics of evolution in recurrent meningioma and, ultimately, could provide insight into natural history. These results suggest that *TERT*p mutations arise later in tumor evolution in progressive meningiomas, consistent with similar observations in other cancers like melanomas or hepatocellular carcinoma [12–14].

**Table 2:** Emergence of *TERT* promoter mutations in high-grade meningioma

	Initial WHO grading	*TERT* promoter status	Recurrent tumor WHO grading	*TERT* promoter status	Number of recurrences
**Pat06**	III	C228T mutation	No recurrence	n.a.	0
**Pat07**	II	wild-type	III	C228T mutation	5
**Pat08**	II	wild-type	III	C250T mutation	6
**Pat24**	II	wild-type	III	C250T mutation	1
**Pat25**	II	wild-type	III	C228T mutation	1
**Pat26**	I	Unknown	II	C250T mutation	3

f: female m: male, n.a.: not applicable, WHO: World Health Organization.

### Spatial intratumoral heterogeneity in *TERT*p-mutant meningiomas

From the perspective of tumor cell phylogeny, later emergence of a subclonal mutational event typically manifests as spatial heterogeneity within a bulk tumor mass. For instance, increased cytogenetic abnormality in recurrent meningioma has recently been demonstrated using array comparative genomic hybridization [15]. However, to date there has been no targeted assessment of intratumoral spatial heterogeneity of meningioma driver genes in progressive samples, due to the fact that most analyses were performed on single tissue samples from individual patients. We therefore scored *TERT*p status at spatially distinct sites in selected recurrent meningiomas in our cohort.

**Figure 2:**
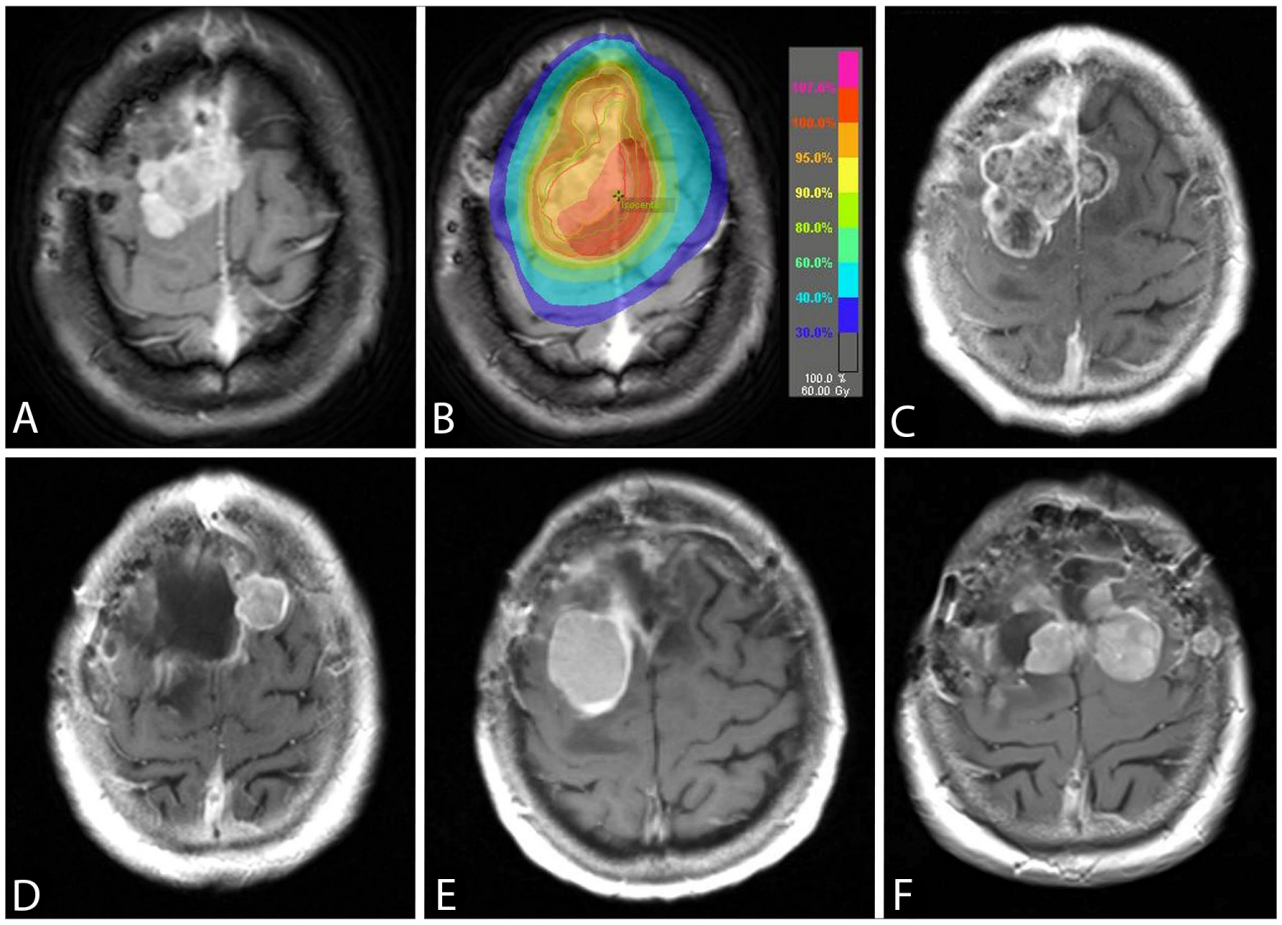
An axial post-gadolinium T1-weighted image showing the *TERTp* wild-typeconvexity WHO grade II meningioma (case Pat08) **(A)**. A radiation therapy with 60 Gy was applied after a subtotal tumor resection **(B)** and the patient was progression-free for 12 months. The follow-up MRI showed a progressive tumor **(C)** and the pathological diagnosis was consistent with an anaplastic meningioma WHO grade III with a new developed *TERTp* mutation. While the recurrence in **(D)** was proven to be *TERTp* wild-type, the subsequent recurrence **(E)** was *TERTp*-mutant. At the time of last surgery **(F)**, the *TERTp* mutation was detected in the right-sided, but not in the left-sided meningioma. The patient passed away 22 months after the first emergence of *TERTp* mutation due to progressive disease.

**Figure 3:**
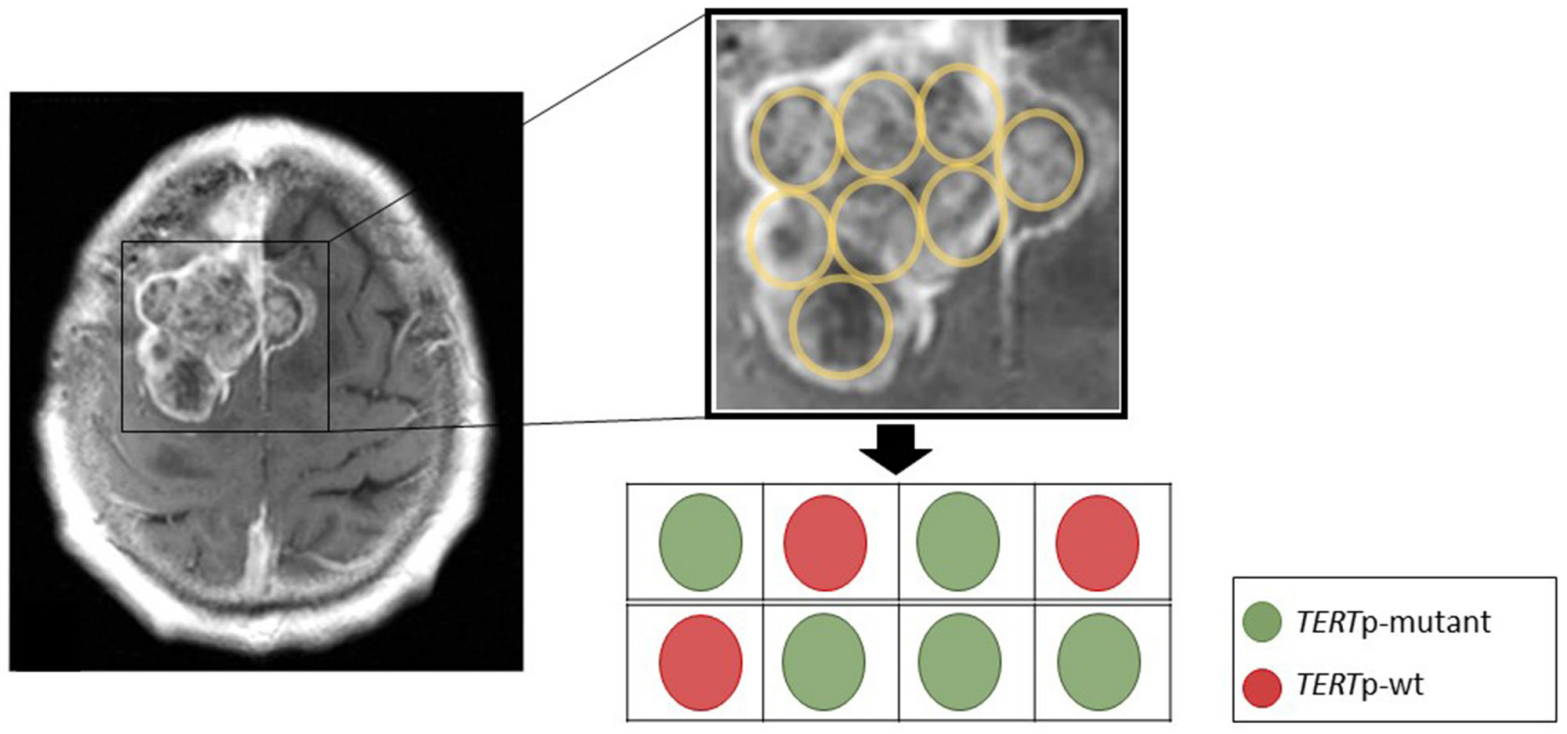
Spatial representation of the tumor from Figure 2C for TERTp mutation status The sequencing of TERTp mutation on DNA extracted from 8 different paraffin embedded tumor blocks from the surgical resection specimen to provide a widely spread and independent geographical separation for the sampling. Although we are not able to directly localize these block specimens with the MRI scan, sequencing revealed a mutation in 5 out of 8 samples, providing evidence for spatial intratumoral heterogeneity in TERTp mutant meningiomas.

In two patients who underwent surgery for recurrent tumors at distinct locations, we detected the *TERT*p mutation in only one of two sites (Figure 2). To further explore intratumoral heterogeneity, we sequenced the *TERT*p in further 8 separate specimens of geographically distinct regions taken from both meningiomas. *TERT*p mutations were present in 5 of the 8 additional samples (Figure 3). Along with the observation that not all recurrent tumors have *TERTp* mutations, this finding suggests ongoing subclonal selection during the evolution of progressive meningiomas, resulting in at least two distinct meningioma cell subpopulations (*TERT*p wild-type and *TERT*p-mutant).

### Presence of detectable mutant *TERT*p in progressive meningiomas confers a poor prognosis

**Figure 4:**
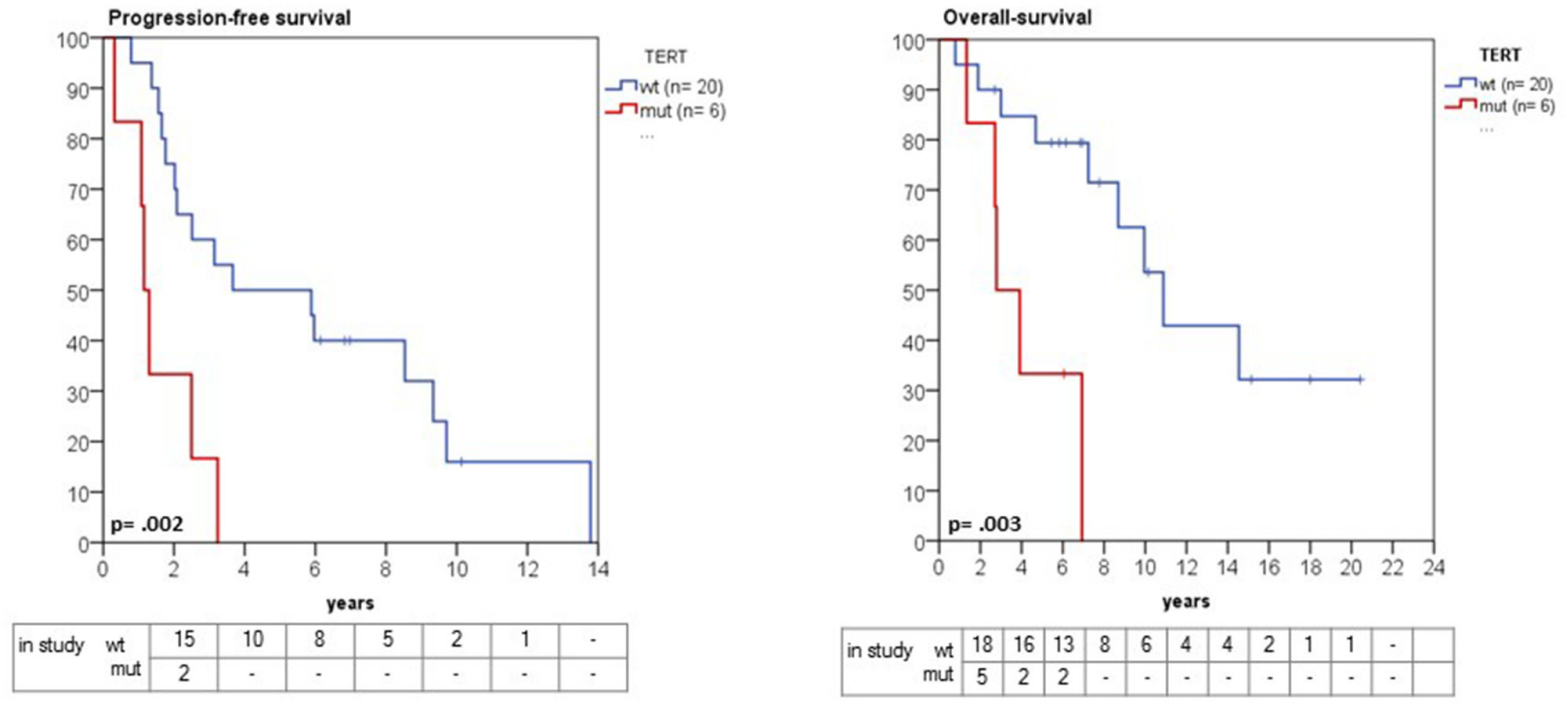
Kaplan-Meier estimates of progression and overall survival in grade II and III meningioma in relation to *TERT*p mutation status Patients with a *TERT*p mutation had a significantly shorter progression-free survival (1.1 years, 95% CI 0.8 - 1.4 versus 3.6 years, 95% CI 0.3 - 9.6, p= 0.002). Likewise, overall survival was significantly shorter in *TERT*p-mutant compared with wild-type patients (2.7 years, 95% CI 0.9 – 4.5 versus 10.8 years, 95% CI 7.8 -12.8, p=0.003).

In a recently published study, Sahm et al. found a strong association between *TERT*p mutations and time-to-recurrence [8], but were not able to evaluate overall survival. With the comprehensive follow-up in our patient cohort, we found that patients with emergence of *TERT*p*-*mutant meningiomas was associated with a significantly shorter OS than their *TERT*p wild-type counterparts, when measured from the time of initial diagnosis (2.7 years, 95% CI 0.9 – 4.5 versus 10.8 years, 95% CI 7.8 -12.8, p=0.003) (Figure 4). In addition, patients with a *TERT*p mutation had a significantly shorter progression-free survival (1.1 years, 95% CI 0.8 - 1.4) in comparison with *TERT*p wild-type patients (3.6 years, 95% CI 0.3 - 9.6, p= 0.002) (Figure 4). In a multivariate Cox regression model, *TERT*p mutation emergence showed significant independent association with poor prognosis (hazard ratio 12.74, 95% CI: 1.77 – 91.8, p= 0.012) (Table 3). Other clinical parameters such as age, gender, tumor localization, initial WHO grade, number of recurrences or adjuvant therapy were not independent prognostic factors in our cohort, although it is possible that there are other clinical variables which may account for the differences in outcome.

**Table 3:** Stepwise backward logistic regression for overall survival

	Hazard ratio (HR)	95% CI	p-value
Age	0.96	0.95 -1.03	0.79
Gender	1.31	0.36 -4.73	0.68
Tumor localization (skull base/ non-skull base)	1.18	0.32 – 4.33	0.96
Initial WHO grade (I/II vs. III)	4.34	0.38 – 48.6	0.23
*TERT*p mutation	12.74	1.77 – 91.8	**0.012**
Number of recurrences	1.34	0.2 - 6.2	0.71
Adjuvant radio- or chemotherapy	1.02	0.057 – 18.52	0.98

### Frequency of *TERT*p mutations in a subset of radiation-associated meningiomas

Five of 6 patients with *TERT*p mutation had received radiation before surgical resection of the specimen demonstrating the mutation, raising the possibility of a causal association between receipt of radiation and emergence of *TERT*p mutation. To test the hypothesis that *TERT*p mutations might be enriched in post-radiation meningiomas, we performed a *TERT*p mutation analysis in a separate cohort consisting of 19 patients with post-radiation meningiomas (n=19 patients, total of 24 samples). On initial diagnoses, these meningiomas spanned all WHO grades (4 grade I, 12 grade II and 3 grade III). However, *TERT*p mutation was detected only in two patients (10.5%). Both had received radiation in childhood, one patient for an orbital tumor, and a second for a pilocytic astrocytoma. Thus, we did not detect a markedly elevated rate of *TERT*p mutation in meningiomas arising after receipt of radiation.

### TERT rearrangements in malignant meningiomas

**Figure 5:**
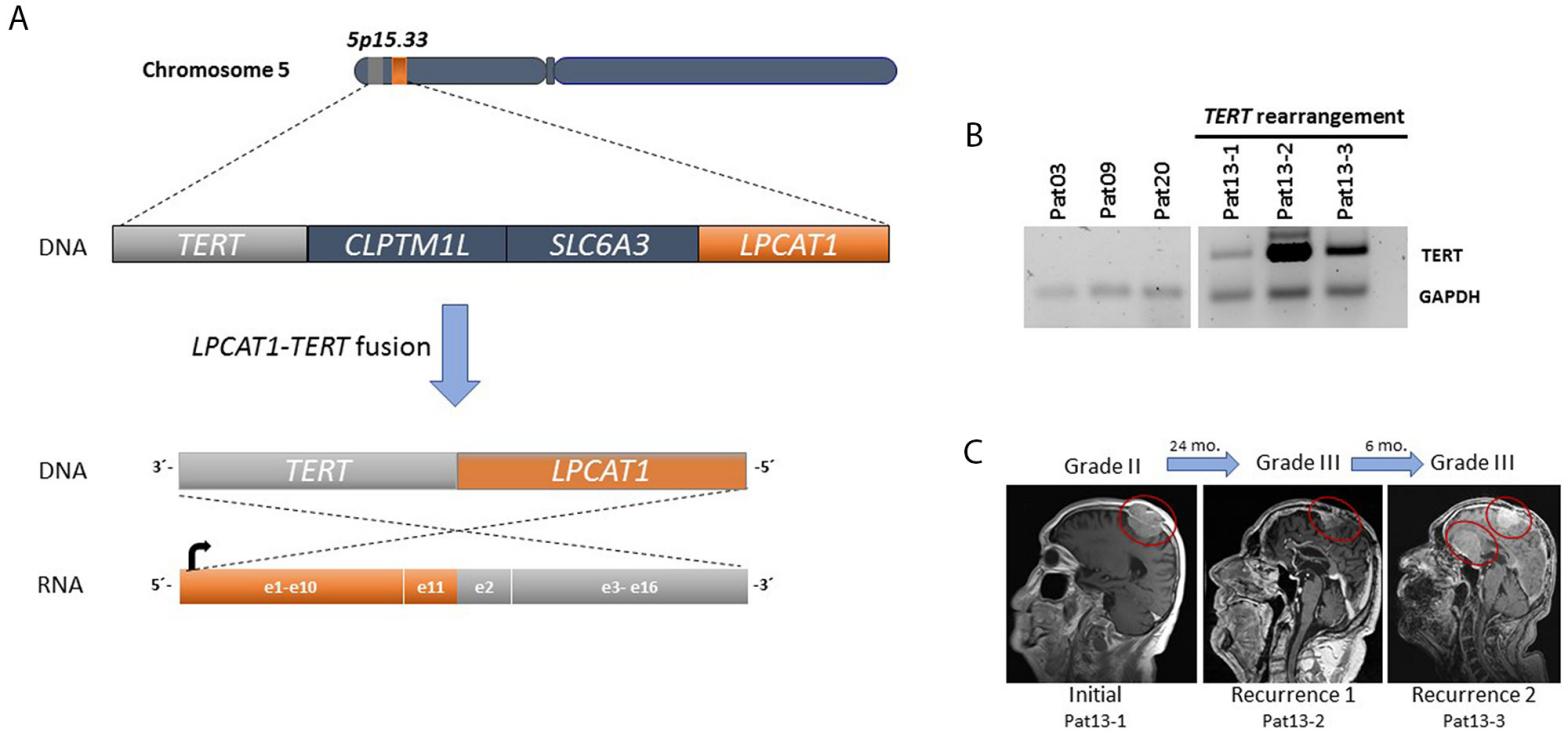
**(A)** A fusion was detected between exon 11 of *LPCAT1* (orange) and exon 2 of *TERT* (gray) within chromosome 5p15. 33. **(B)** *TERT* expression (reverse transcriptase PCR) in three subsequent samples of patient 13 with *TERT* rearrangements compared to three samples from patients without *TERT* alterations. *TERT* was amplified in all consecutive samples. *GAPDH* was used as a control. **(C)** Representative MRIs for tumor growth history for patient 13.

*TERT* rearrangements have been described in several neoplasms and are usually associated with increased *TERT* expression [5]. To assess whether *TERT* gene rearrangements were present in progressive higher-grade meningiomas, we performed targeted RNA fusion gene analysis in *TERT*p wild-type meningioma cases (n=28) using Anchored Multiplex PCR (AMP). We identified a novel chromosomal rearrangement involving *TERT* in a recurrent WHO grade III meningioma sample (patient 13, Table 1). The fusion involved the juxtaposition of exon 11 of *LPCAT1* and exon 2 of *TERT* within chromosome 5p15.33 (Figure 5). The remaining samples did not have detectable TERT exon 2 fusion transcripts.

### Progressive meningiomas lack *ATRX* and *DAXX* mutations

As an alternative to upregulation of *TERT* expression, cancers can also use the ALT pathway for telomere maintenance, associated with inactivating mutations in either the *ATRX* or *DAXX* genes [16]. Therefore, we screened 18 samples of our *TERT*p-wild-type (n=15) and *TERT*p-mutant (n=3) malignant meningioma for mutations in both genes using targeted sequencing. None of the samples showed *ATRX*, *DAXX* mutations or deletions. In addition, we performed *ATRX* immunohistochemistry from meningiomas in our cohort at different stages during progression (n= 42). In one case, *ATRX* expression was lost by IHC, however, targeted analysis by SNaPshot NGS did not reveal mutations or deletions in the *ATRX* gene. Together with a recent report of no loss of *ATRX* or *DAXX* expression by IHC in 58 grade II and III meningiomas [16], these results provide evidence that the ALT pathway is not a frequent mechanism for telomere maintenance in progressive higher-grade meningiomas.

## DISCUSSION

We demonstrate that mutations of *TERT*p are highly-prevalent in progressive higher-grade meningiomas, found in up to 25% of cases. These findings support similar observations reported by Goutagny et al., who found an increased incidence of *TERT*p mutations in patients with meningiomas that underwent malignant progression [3]. However, Goutagny et al. showed in all patients with multiple specimens that the *TERT*p mutation was present in the initial low-grade tumor [3]. In contrast, we reveal the emergence of *TERT*p mutations during the progression interval, providing evidence for the outgrowth of subpopulations of tumor cells during the evolution of these tumors.

Similarly, a recent integrated genomic analysis detected *TERT*p mutations in 4/31 recurrent atypical meningioma samples, but not in any of 110 first-diagnosed, non-recurrent grades I and II meningiomas [9]. The authors speculated that acquisition of an activating *TERT*p mutation could be an essential step in formation of atypical meningiomas [9]. As a proof of this later clonal emergence, our study demonstrates *TERT*p mutations were heterogeneously detected in geographically distinct sites within recurrent bulk tumor mass, providing critical evidence in support of this model. Further consistent with this model of accelerated tumor progression, we show that *TERT*p-mutant meningiomas are associated with a significantly shorter overall survival than their *TERT*p wild-type counterparts.

In addition to the *TERT*p mutations, we identified for the first time in meningioma a fusion that involves the *TERT* gene (*LPCAT1:TERT*). In this case, the patient (pat13) had a meningioma grade II that progressed to grade III within two years (Figure 5). Interestingly, *LPCAT1-TERT* fusions has been described in lung adenocarcinoma [17] and in a pediatric hepatocellular carcinoma [18]. *TERT* rearrangements are a common genetic alteration in high-risk neuroblastomas and are associated with increased *TERT* expression and a poor prognosis [19].

Taken together, these findings indicate that meningiomas likely give rise to more malignant clones during an ongoing evolutionary process, underscoring the crucial role of genetic diversity in tumor cell evolution. Furthermore, this heterogeneity might explain the variable detection frequency of *TERT*p mutation that has been reported in atypical meningiomas [9]. Indeed, the high treatment-failure rate of progressive meningiomas may be due to this increased heterogeneity allowing subclonal escape from adverse selection pressures such as hypoxia, metabolic stress, chemotherapy, or radiotherapy [20].

Our findings have implications for understanding the biology of a subset of progressive higher-grade meningiomas, providing important insight into the complexity of intratumor heterogeneity. Distinct molecular etiologies drive progressive higher-grade meningiomas, overlaid on the known genomic alterations in newly-diagnosed benign tumors. These differences highlight the potential role of *TERT* alterations as a clinical biomarker allowing for the stratification of patients in the recurrent setting for clinical trials in progressive higher-grade meningioma. Sensitive techniques for the detection of *TERT*p-mutant subclones may allow for the early diagnosis of clinically-aggressive meningioma, a long-sought goal of molecular analyses of this disease.

## MATERIALS AND METHODS

### Patient and tumor characteristics

Twenty-six patients with treatment-resistant meningiomas formed the study cohort, with specimens available from initial diagnosis and sequential recurrences (n=64 tumors). Patient demographic characteristics are shown (Table 1). Tumor locations included 11 convexity, 9 skull base, two infratentorial and one intraventricular meningiomas; MRI imaging at time of initial diagnosis was not available for two patients. Three patients presented initially with a WHO grade I meningioma, while 13 patients had a grade II meningioma at initial diagnosis, and 10 patients had a *de novo* grade III meningioma at first surgery. Most patients (n= 19) in this study had recurrent surgery in addition to the initial diagnostic procedure, during the median follow-up of 11.3 years (range 4.4 – 16.8 years), undergoing an average of 3 surgeries (range 1-8). In one case upfront radiotherapy was performed, whereas the majority (21/26, 80.7%) received radiotherapy during the course of treatment after the initial surgery. In two cases, the initial surgical specimen was unavailable, as that surgery had been performed at an outside hospital. Overall, 19 patients had one or more recurrent meningioma specimens available, with the initial, pre-treatment tumor sample available for these cases, except for patient Pat26.

Tumor and blood samples from patients were obtained from the Departments of Neurosurgery at the University Hospital Dresden and the Massachusetts General Hospital in Boston under established Institutional Review Board approvals for genetic studies. When fresh tumor tissue from surgical specimens was available, it was immediately frozen at −80 °C. When frozen tissue was not available, formalin-fixed, paraffin-embedded tissues were used for DNA extraction. Control slides stained with hematoxylin and eosin were reviewed by a neuropathologist to assure a tumor cell content of at least 80% for nucleic acid extraction. DNA isolation was performed using the QIAmp DNA Mini Kit 50 (Qiagen, Hilden, Germany).

### *TERT* promoter mutation

The *TERT*p was assessed either by amplification using Sanger sequencing performed using ABI Prism 3730 DNA Analyzer or using the fluorescence PCR technique previously described [21]. Mutations between the residues of −124 (1295228) to −146 (1295250) bp from the ATG start site in the *TERT*p were scored, as described previously by Killela et al. [22].

### SNaPshot Next Generation Sequencing Archer® FusionPlex®

Specimens were subjected to additional analysis utilizing SNaPshot, a hybrid capture based method for single nucleotide variant (SNV) and insertion/deletion (indel) detection in genomic DNA, and Archer® FusionPlex®, an anchored multiplex polymerase chain reaction (AMP) technique by ArcherDx (Boulder, CO) for fusion detection in RNA [23]. SNaPshot targets 108 genetic loci frequently mutated in 15 cancer genes, including *TERT*p, *ATRX*, and *DAXX*. AMP using the Archer® FusionPlex® Solid Tumor Kit detects gene rearrangements involving over 50 genes, including unknown fusion gene partners such as novel *TERT* exon 2 fusion transcripts [23].

### Reverse transcriptase PCR

cDNA was synthesized using 10 μg of total RNA with the High-Capacity cDNA Reverse Transcription Kit according to the manufacturer’s instructions. *TERT* was amplified using primer sets forward sequence 5’-CTGCAGGCGTACAGGTTTC-3’ and reverse 5’-GTGTCGAGTCAGCTTGAGCA-3’. GAPDH primers were used as control with the forward sequence 5’-GTCAGCCGCATCTTCTTT-3’ and reverse 5’-CGCCCAATACGACCAAAT-3’. The amplified products were separated on a 1.5% agarose gel.

### *ATRX* immunohistochemistry

Immunohistochemistry (IHC) was performed with a polyclonal rabbit antibody against ATRX (dilution 1:100, Abcam, AB97508), using published protocols [24]. Two independent neuro-pathologists evaluated all IHC results (E.A.W. and M.A.). Nuclear staining was considered positive for evaluation. Cases with more than 10% of tumor cells staining positively were considered “retained”. Endothelial cells, cortical neurons and infiltrating inflammatory cells were generally positive and served as internal positive controls.

### Clinical data

Demographic, treatment and follow-up data were retrospectively collected. Progression free survival (PFS) was calculated from the initial diagnosis until radiographic tumor progression as determined by the treating physician. Overall survival (OS) was defined as the interval from the day of first surgery until death, with data censored at the last available date of follow-up. All patients’ data were updated on December 10, 2016.

### Statistical analysis

The Kaplan-Meier technique was used to estimate PFS and OS and significant differences were analyzed by the log-rank test using the statistical software SPSS. The Mann-Whitney *U* and Fisher’s exact tests were used to test for association of clinical variables and *TERT* alterations. *P* < 0.05 was considered as statistically significant.

## Abbreviations

ALT: alternative lengthening of telomeres
CTx: chemotherapy
DAXX: death domain associated protein
OS: overall survival
PFS: progression-free survival
RTx: radiation therapy
TERTp: telomerase reverse transcriptase promoter
WHO: World Health Organization
